# Real-time ultrasound-guided sacral plexus block combined with mild sedation for hemorrhoidectomy and hemorrhoidal artery ligation in a patient with amyotrophic lateral sclerosis: a case report

**DOI:** 10.1186/s13256-024-04493-4

**Published:** 2024-04-24

**Authors:** Yan Li, Qianhui Hu, Qian Wang, Taotao Liu, Min Gao

**Affiliations:** Department of Anesthesiology, Guangyuan Central Hospital, Guangyuan, 628000 China

**Keywords:** Amyotrophic lateral sclerosis, Sacral plexus block, Dexmedetomidine, Case report

## Abstract

**Background:**

Patients with amyotrophic lateral sclerosis present perioperative challenges for clinical anesthesiologists for anesthesia-associated complications.

**Case presentation:**

A 54-year-old Han woman with a 2-year history of amyotrophic lateral sclerosis was scheduled for hemorrhoidectomy and hemorrhoidal artery ligation. We performed real-time ultrasound-guided sacral plexus block with dexmedetomidine under standard monitoring. The anesthesia method met the surgical demands and avoided respiratory complications during the procedures. There was no neurological deterioration after the surgery and 3 months after, the patient was discharged.

**Conclusions:**

Real-time ultrasound-guided sacral plexus block combined with mild sedation may be an effective and safe technique in patients with amyotrophic lateral sclerosis undergoing hemorrhoidectomy and hemorrhoidal artery ligation.

## Background

Amyotrophic lateral sclerosis (ALS) is a progressive neurodegenerative disease that is characterized by the degeneration of both upper motor neurons and lower motor neurons, leading to motor and extramotor symptoms, such as dysphagia, dysarthria, respiratory insufficiency, muscle cramps, spasticity, muscle weakness, muscle atrophy, cognitive impairment, and behavioral impairment [1]. Adequate perioperative management of patients with ALS is necessary. Patients with ALS are more susceptible to neuromuscular blocking agents and other anesthetics than other patients [2]. General anesthesia may inhibit respiratory function and the swallowing reflex and exacerbate the weakness of respiratory muscles, which leads to regurgitation and hypoxia [3]. Neuraxial blockade is an alternative method for patients with hemorrhoidectomy and hemorrhoidal artery ligation, but it may exacerbate ALS symptoms because of needle injury and drug toxicity [4]. Although peripheral nerve blockades are relatively contraindicated because of the trauma of puncture, nerve injury, nerve ischemia, and local anesthetic toxicity, recent studies have shown that peripheral nerve blockades provide satisfactory analgesia and perioperative safety in patients with ALS [5–7]. Thus, we present a case of a patient with ALS who successfully underwent hemorrhoidectomy and hemorrhoidal artery ligation under real-time ultrasound-guided sacral plexus block combined with mild sedation. The patient and her husband (caretaker and power of attorney) provided written consent publication of case report or identifying information/images in an online open access publication.

## Case presentation

A 54-year-old female patient (body weight 58 kg; height 162 cm; ethnicity: the Han nationality) with a 2-year history of ALS was scheduled for hemorrhoidectomy and hemorrhoidal artery ligation due to hemorrhoids. There were symptoms of muscle weakness of the right lower limb for 3 years that had aggravated for 1 year and inflexibility in both hands for 1 year. She was diagnosed with ALS 1 year ago. She was also diagnosed with mild cognitive impairment according to the Minimum Mental State Examination (MMSE) score. Physical examinations showed that her pulmonary function was normal, and she had no difficulty speaking or swallowing. There was no sensory abnormality. To avoid possible damage to neuromuscular function and respiratory depression, we decided to administer real-time ultrasound-guided sacral plexus block combined with mild sedation for anesthesia.

**Fig. 1:**
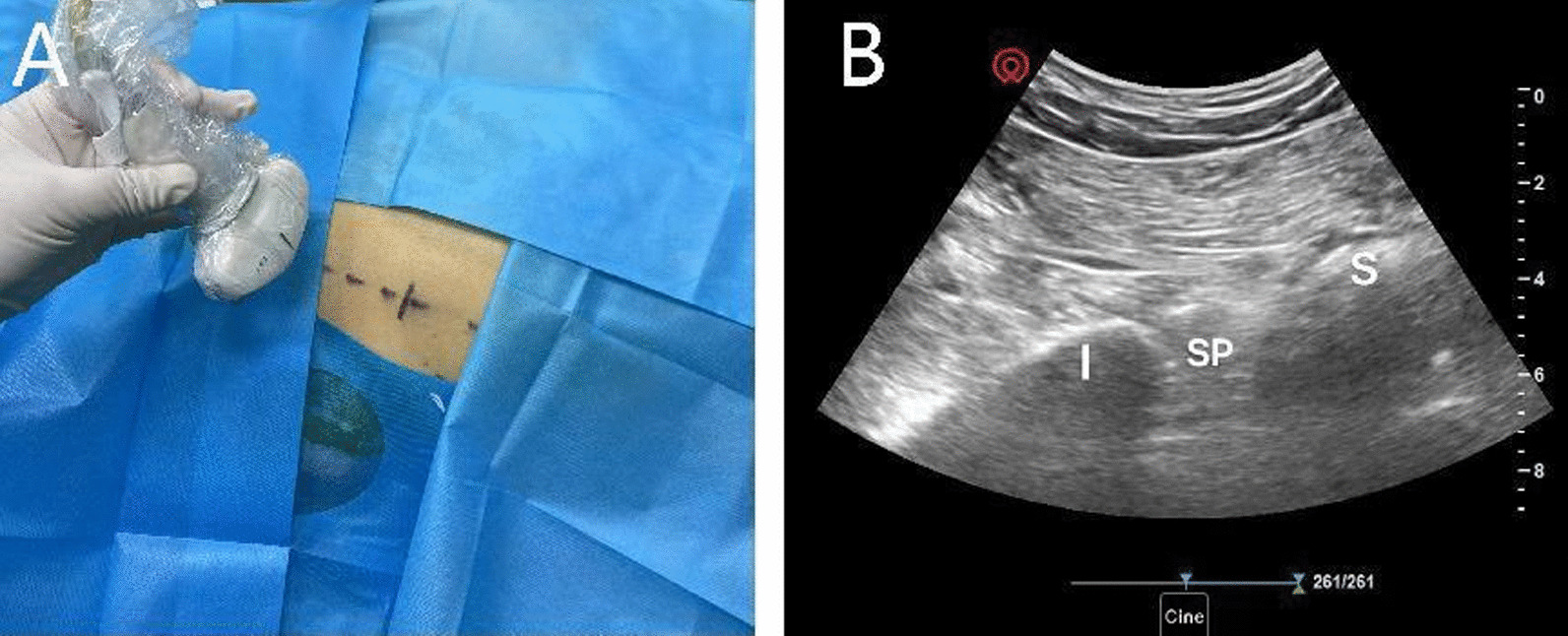
Approach of real-time ultrasound-guided sacral plexus block. *I* ilium, *SP* sacral plexus, *S* sacrum

After transferring the patient into the operating room, standard monitoring was established, including noninvasive arterial blood pressure, pulse oximetry, and electrocardiogram (ECG) monitoring. The patient was placed into the prone position. Real-time ultrasound was used to confirm the location of the sacral plexus. First, the ultrasonic probe was placed at the midpoint between the greater trochanter of the femur and the posterior superior spine of the iliac, with the lower margin of the inner half level. At this time, an oblique linear high-echo shadow of the ilium was seen on the ultrasound image. The probe was then slid inward and downward to see the image of the inner sacrum and the outer ilium, and the high-echo sacral plexus was located between the ilium and the sacrum (Fig. 1A, B); 15 mL of 1% lidocaine was injected with an in-plane technique guided by real-time ultrasound on both the left and right sides. The procedure was performed by an experienced anesthetist using aseptic techniques. The patient was then placed into the supine position. Intravenous infusion of dexmedetomidine was administered for sedation at 1 µg/kg/hour for 10 minutes and continued at 0.3 µg/kg/hour along with 2 L/minute O_2_ via a face mask. The operation began after confirming that the perineum area had no pain response.

The operation time was 30 minutes. There was no pain or discomfort intraoperatively. In case of the occurrence of anesthesia-associated complications, the patient was monitored in the post-anesthesia care unit for 1 hour after the surgery. The patient was discharged on the fifth day after the surgery. There was no obvious deterioration of ALS symptoms after the surgery or 3 months after the patient was discharged.

## Discussion and conclusions

ALS is a fatal neurodegenerative disease primarily affecting upper and lower motor neurons. In this case, the patient was suspected of having limb-onset ALS, presenting with symptoms in the upper and lower limbs. In addition, there was mild cognitive impairment. Currently, there is no agreement on the anesthetic method of patients with ALS undergoing hemorrhoidectomy and hemorrhoidal artery ligation. The concerns about the patient were mainly regarding minimizing the effects on the function of motor and cognition, meeting the analgesic requirement, and preventing the perioperative risk of respiratory, bulbar, and autonomic nervous system complications. Hence, real-time ultrasound-guided sacral plexus block combined with mild sedation was decided upon as the best option for the patient after discussion of the multidisciplinary team.

Generally, general anesthesia, neuraxial blockade, and peripheral nerve block can meet the surgical requirements of patients with hemorrhoids. However, for patients with ALS, the anesthetic goal is to satisfy the analgesic requirement with the least variety of drugs, the smallest amount of drugs, and the simplest techniques without affecting neurological function. The research showed that general anesthesia can be used in patients with ALS, but it has a risk because many general anesthetics have different degrees of respiratory depression, and mechanical ventilation is often needed [8]. Muscle relaxants such as depolarizing neuromuscular blockers are strongly avoided, and nondepolarizing neuromuscular blockers should be used with caution [2, 9]. Spinal and epidural anesthesia are conflicting according to previous studies. Although it is safely used in patients with ALS, patients with neurological diseases experience worsening neurologic symptoms and neurological sequelae after spinal anesthesia [10–12]. In terms of avoiding respiratory failure, protecting laryngeal reflexes, and maintaining hemodynamic stability, peripheral nerve blocks have advantages over other anesthesia methods [7].

The sacral plexus is considered to be formed anatomically by the lumbosacral trunk and the ventral rami of the first three sacral spinal nerves and descending portion of the fourth sacral spinal nerve, which innervates the skin and muscles of the gluteal region, posterior compartment of the thigh, popliteal fossa, posterior and lateral compartments of the leg, and dorsum of the foot [13]. Ultrasound-guided regional nerve block greatly increases the accuracy of local anesthetic administration around the nerve, and the advantages of ultrasound guidance include visualization of anatomic structures, reduction of local anesthetic requirements, improvement in anesthetic block quality, and prevention of fatalities [14]. In addition, real-time ultrasound guidance can provide anatomical positioning images of the nerve and the dynamic trajectory of the puncture needle. Dexmedetomidine is a highly selective α2-adrenergic receptor agonist with sedative, analgesic, anti-anxiety, and inhibitory effects on sympathetic nervous activity, with minimal respiratory depression [15]. Clinical trials have demonstrated that dexmedetomidine has a protective effect on the cognitive function of patients with mild cognitive impairment [16, 17]. Therefore, real-time ultrasound-guided sacral plexus block combined with dexmedetomidine sedation seems to be an effective and safe technique for patients with ALS with hemorrhoids.

In summary, this case presents the use of real-time ultrasound-guided sacral plexus block combined with mild sedation in a patient with a 2-year history of ALS and subsequent hemorrhoidectomy and hemorrhoidal artery ligation. The anesthetic strategy successfully met the demands of the surgery and avoided respiratory and neurological complications. However, the efficacy of our strategies may be affected by selection bias because of the nature of case reports. Further studies are needed for this high-risk patient population.

## Data Availability

All data generated or analyzed during this study are included in this published article.

## Abbreviations

ALS: Amyotrophic lateral sclerosis
MMSE: Minimum Mental State Examination
ECG: Electrocardiogram
